# “It’s sink or swim for us”: The lived experiences of Filipino nurses in the UK during the COVID-19 pandemic

**DOI:** 10.1016/j.qrmh.2025.100002

**Published:** 2025-03-18

**Authors:** Denise Borbolla, Ohemaa Nkansa-Dwamena

**Affiliations:** Department of Psychology, City St George’s, University of London, School of Health & Medical Sciences, London, United Kingdom

**Keywords:** Filipino, Nurses, Healthcare workers, Mental health, COVID-19 impacts, Ethnic minority research, Lived experiences

## Abstract

**Background:**

The United Kingdom has recruited Filipino nurses since the late 1990s to meet the country’s healthcare needs. Currently, over 40,000 Filipinos are working in the National Health Service, and it is suggested that 36 % of all known healthcare worker (HCW) deaths from COVID-19 within the first two-month period (March and April 2020) were Filipinos, despite accounting for 8 % of the NHS nursing workforce. There was a clear disparity in social media exposure between the celebrated heroism of Filipino HCW and the coverage of disproportionate death rates within the Filipino HCW community in the UK. This study aimed to explore the lived experiences of Filipino nurses in the UK during the COVID-19 pandemic.

**Methods:**

A qualitative study was conducted using interpretative phenomenological approach (IPA). Six nurses were recruited using purposive and snowball sampling, and interviews were transcribed verbatim and analyzed using IPA.

**Findings:**

Drawing on interview data, two themes are presented: *inescapability and relentlessness of COVID-19* and *“It’s sink or swim”: psychological welfare*.

**Interpretation:**

Filipino nurses experienced the COVID-19 pandemic as an all-consuming phenomenon, as they were perceived as the embodiment of threat and placed at disproportionate risk perpetuated by racial, systemic, and political factors. Despite this, they had no choice but to battle through, engaging in culturally specific ways of coping.

## Introduction

Utilizing interview data with six Filipino healthcare workers (HCWs), this paper explores and describes their complex and nuanced experiences of being on the front line of the COVID-19 pandemic in the United Kingdom (UK). Declared as a global pandemic by the World Health Organization (World Health Organization, 2020) on March 11, 2020, COVID-19 had a monumental effect on all aspects of life. The impact cannot solely be measured by the vast death rate impacting all communities; disparities in access to healthcare, education, and work as a result of pre-existing political, sociocultural, and structural inequalities must also be considered (Blundell et al., 2020, Hayward et al., 2021). HCWs have been at the forefront of the COVID-19 pandemic in their face-to-face treatment and care of patients, and therefore, HCW have been exposed to greater risks compared to the general, non-clinical professions (Spoorthy et al., 2020). Filipino HCWs, in particular, have been disproportionately impacted during the COVID-19 pandemic, as it is suggested that 36 % of all known HCW deaths from COVID-19 within the first two-month period (March and April 2020) were Filipinos (Cook et al., 2020); the detrimental impact on the Filipino HCW community was further compounded by an increase in targeted racism and xenophobia (Galam, 2020).

The impacts of COVID-19 on the HCW community have been documented thoroughly, with research published within the first few months following the lockdown in the UK (Maben and Bridges, 2020, Vindrola-Padros et al., 2020). Wanigasooriya et al. (2020) conducted one of the first studies exploring this phenomenon, using a cross-sectional survey of 2638 participants which took place between June 5 and July 31, 2020; this study demonstrated that one-third of the hospital HCWs reported clinically significant levels of anxiety and depression, while one-quarter reported clinically significant post-traumatic stress disorder (PTSD) levels. Similarly, Gilleen et al. (2021) assessed the mental health well-being of 2772 HCWs shortly after the first national lockdown in the UK, revealing higher levels of distress and worsening mental health among frontline HCWs compared to non-frontline HCWs and noting several controllable factors such as lack of personal protective equipment (PPE), higher workloads, and lack of workplace preparation, all of which attributed to their decreased mental health.

In contrast to the extensive body of research focusing on HCWs during COVID-19, there is a notable dearth of literature concerning HCWs from ethnically marginalized backgrounds; this is problematic, given the evidence suggesting that people from ethnically marginalized communities are more at risk for severe and long-term impacts of COVID-19 (Ayoubkhani et al., 2021; Bhatia, 2020), long-term adverse mental health impacts (Moorthy and Sankar, 2020, Iob et al., 2020), and disproportionate death rates (Public Health England, 2020). A literature search identified research relating to Black, Asian, and minority ethnic (“BAME”) HCWs in the UK; however, this is problematic as it homogenizes the experiences of intersectionality. The grouping of many different ethnicities into one umbrella term conflates issues to do with race that are not the same for everyone (Ali, 2020). Additionally, it allows for government and businesses to minimize each group’s experiences and perpetuates systemic and institutionalized racism. Examples of this are UK and NHS systems not recognizing Filipinos as a separate ethnic group (Galam, 2020) and Public Health England’s COVID-19 inequalities report which failed to highlight that one in four NHS deaths were Filipinos (PHE, 2020).

The UK has a longstanding history with Filipino nurses, relying on their recruitment since the 1990s to meet the country’s healthcare needs (Hayakawa, 2020). At present, over 40,000 Filipinos are working in the NHS, accounting for the second largest ethnic group in the nursing workforce (Baker, 2022). The reliance on Filipino HCW is imperative as seen in the recruitment of Filipino nurses during the height of the COVID-19 pandemic and amidst lockdown measures, as it was reported that 80 nurses joined Great Ormond Street Hospital to help fill in longstanding vacancies within the trust^1^ (GOSH, 2021). Despite the ever-growing presence of Filipinos in the NHS, they account for just over two percent of the entire NHS workforce and eight percent of the nursing workforce, yet have the highest death rates within the NHS workforce’s ethnic minoritized community (Brown, 2020, Cook et al., 2020). Cook et al. (2020) reported that 36 % of all known HCW deaths from COVID-19 within the first two-month period (March and April 2020) were Filipinos, and, 25 % of NHS deaths were Filipinos (Baker, 2022). Furthermore, this disproportionate impact on the Filipino HCW community is compounded by an increase in targeted racism and xenophobia (Galam, 2020). At the time of literature search, only four studies pertaining to Filipino nurses’ experience during COVID-19 were found; two of which were conducted in the UK (Galam, 2020, Humi, 2022), one in Dubai (Pogoy & Cutamora, 2021), and one in the US (Nazareno et al., 2021).

**Fig. 1:**
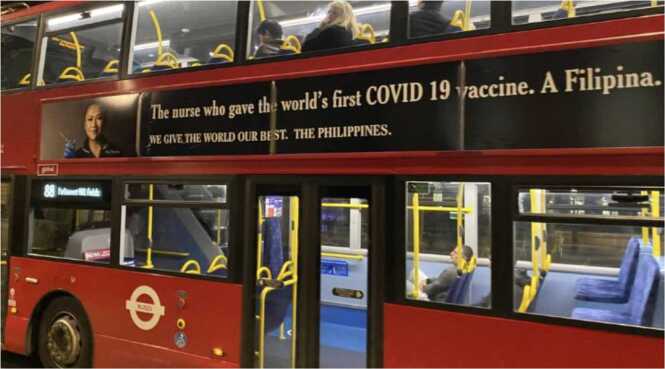
London bus advert with a recruitment campaign for Filipino nurses. Note. Photo from May Parsons, X. May 7 2023 https://x.com/mayparsons/status/1655276389354942465.

Galam (2020) identified the intensity of the racialization of COVID-19 within the Filipino HCW community. Filipino HCW became the epitome of the “hero” carer, highlighted through media coverage emphasizing Filipino HCWs’ selflessness and heroism, including May Parsons, a Filipino nurse who was the first person in the world to deliver a COVID-19 vaccine to a patient. This event became highly publicized, and Parsons became the face of Filipino HCW recruitment across the UK, with advertisements seen on London buses (Fig. 1).

Simultaneously, Filipino HCWs were being seen as virus-carriers, which were racist viewpoint exacerbated by the significant homogenization of Asians (Wang et al., 2021) and increase in anti-Asian hate crimes occurring within Western societies. This also led to a significant increase of physical and verbal abuse against the Filipino HCW community (Galam, 2020). Humi (2022) further explored this phenomenon by foregrounding Filipino nursing history in Filipino HCW experiences of COVID-19, showcasing the dualism of the “healthcare hero” and the “manufactured hero” identities; on one level, Filipino HCWs were praised and applauded for their hard work and sacrifice on the front line, and on another level, these traits which have been cultivated specifically for the Western market through centuries of colonialism were exploited particularly during distressing periods such as COVID-19.

Choy (2003) introduced the term “Empire of Care” to encapsulate the longstanding impact of Western colonialism on Filipino nursing history—in particular, the American colonization of the Philippines in the 20th century which followed the 333-year Spanish rule. While the US introduced Western nursing to the Philippines in an attempt to uplift the Filipino people following the long rule of Spain, it also aimed to create a labor force to support the occupational shortage in the US, which ultimately created racialized hierarchies, as Filipino bodies became socially constructed as “weak, diseased and therefore racially inferior” (Choy, 2003, p. 21). The US initiated this transnational labor-migration export (Espiritu, 2005) in the early 1950s, and the UK followed suit in the early 1990s. Today, many Western countries rely on the Filipino HCW labor force to support their healthcare systems, making the Philippines the world’s largest supplier of nurses in the world (Ladrido-Ignacio & Versoza, 2011; Lorenzo et al., 2007).

The paucity of research conducted on Filipino nurses significantly contrasts with research done on HCW during the COVID-19 pandemic, as the terms “COVID-19” and “healthcare workers” generated over 101,860 research articles (Dimensions, 2023). Given the disproportionately high Filipino death rate within the HCW workforce (Galam, 2020, Nazareno et al., 2021), it is worrying that only four studies relating to Filipino nurses during the COVID-19 pandemic have been found in a literature search. As such, this article aims to enrich the limited body of literature by illuminating the idiographic, lived experiences of Filipino nurses who have been essential in the fight against the COVID-19 pandemic in the UK.

## Methods

### Study design and sampling

This article is drawn from a larger IPA study that explored the lived experience of Filipino nurses in the UK during COVID-19 pandemic. The study used a qualitative design method, gathering data from semi-structured interviews focusing on generating meaning and exploring phenomena from the participants’ lived experiences. Participants were recruited using purposive and snowball sampling methods. Specific platforms targeted towards Filipino nurses were approached, and participants were asked to invite friends and colleagues who may be interested in taking part. The sample size was determined by staying true to IPA’s idiographic nature which emphasizes the importance of a small and fairly homogenous sample size of four to 10 participants (Willig, 2013). This study aimed to recruit six to eight participants, and the recruitment process occurred periodically throughout an 18-month period due to difficulties with participant engagement. While there was interest generated from social media posts and research posters circulated on targeted Filipino online communities such as Filipino Nurses Association UK, Filipino UK Nurses, and Kanlungan, many potential participants opted out after initially sharing their interest in the study. This may be explained by the ongoing fears of being seen as a whistle-blower or perhaps the unreadiness of the Filipino nurses as a cohort in processing their lived experiences. Given that the data generated from the participants were rich, nuanced, and emotionally ladened, we agreed to end recruitment after six participants. See Table 1 for participant demographic information.

**Table 1:** Participant demographic information.

Name	Age range	Gender
Anna	33–43	Female
Dominic	44–54	Male
Karla	44–54	Female
Megan	33–34	Female
Carlos	22–32	Male
Christine	44–54	Female

### Ethical considerations

Ethical approval was granted by City St George’s, University of London’s ethics committee. To ensure the confidentiality of participants, identifiable data was redacted on all forms and transcripts, and physical data such as demographic forms were destroyed after being scanned onto an encrypted platform. Participants were informed about the study, its aims, and processes. Participants gave their written consent, and they were informed about their right to withdraw from the study. Given the sensitivity of the topic researched, mitigation strategies were implemented to reduce the risk of psychological harm which included strict inclusion and exclusion criteria and administering the CORE-10 questionnaire (Barkham et al., 2012). Lastly, pseudonyms are used to maintain participant anonymity. (See below for further details.)

### Procedure

Interested participants were invited to a telephone screening call lasting 20 to 30 min. This was used to ascertain whether the participants met the inclusion criteria for the research study: 1) being Filipino by ethnicity, 2) being qualified as a nurse in the Philippines prior their UK move, 3) having moved and worked as a nurse in the UK prior to the COVID-19 pandemic (a year before March 2020), 4) having worked/working as a nurse in the UK during the COVID-19 pandemic, 5) being fluent in English, 6) consenting to being audio recorded, and 7) being between 22 and 67 years old. Additionally, they were assessed for their mental health wellbeing using the CORE-10 questionnaire, as it was anticipated that the interview may bring up sensitive and traumatic memories and experiences. Participants who scored an average of three or more on the CORE-10 were not deemed suitable for the study; however, no participants were screened out Towards the end of the screening call, eligible participants were invited for an interview. Individual interviews lasted 60 to 90 min and took place either in person, in a room located at City, University of London, or online via Zoom using a password-protected computer; a university-approved dictaphone (Olympus Ds 9000) was used to record these interviews. Five participants opted for an online interview, and one participant opted for an in-person interview. Interviews took place over the course of 2022.

An interview schedule consisting of open-ended and non-leading questions was created and used as a guide for the interview consisting of three main sections: 1) introductory questions (“How are you doing/feeling?” and “What drew you towards taking part of this study?”), 2) main questions to explore the experience of working as a nurse (e.g., “What motivated you to become a nurse?,” “What is it like being a nurse?,” “Can you describe to me your experiences as a Filipino nurse caring for patients with COVID-19 in the UK?,” “What is it like being away from the Philippines at the time of the COVID-19 pandemic?,” “Can you tell me what has gotten you through the pandemic so far?”), and 3) any additional questions, reflections, or comments.

The interviews were then transcribed verbatim and analyzed using IPA (Smith et al., 2022) which generated four main group experiential themes. The two themes relevant to the focus of this paper are presented. Participants were debriefed after the interview and were reminded of their ability to withdraw their data from the research up until the point of data analysis, which occurred approximately three months post-interview. Furthermore, participants were given the opportunity to review their transcript before data analysis, which one participant opted for; after reviewing their transcript, the participant did not ask for anything to be removed. Participants were also asked about their emotional state and whether they were experiencing any distress from the interview process. Interviews were conducted in a person-centered manner, focusing on providing a safe and containing space whereby the relationship between the interviewer and participant facilitated the in-depth exploration of meanings attributed to their lived experiences. Lastly, a debrief letter was sent following the session to each participant thanking them for their time, and information for local services, and mental health hotlines were included in case they experienced emotional distress following the interview.

### Reflexivity

It was crucial to consider the lead researcher's perspective shaped by both insider/outsider positioning, as outlined by Dwyer and Buckle (2009). Embracing her “insider” role as a Filipino woman, she held an understanding of Filipino culture, language, family norms, and gendered migration expectations. This familiarity allowed her to establish strong rapport with participants from the outset, fostering a sense of trust. It was also important to acknowledge that her “outsider" position, marked by differences in accent, skin tone, and facial features, may have led participants to hold certain preconceptions that may have impacted their way of relating during the interview. Consultation with the second author served to delve deeper into participants' narratives, reflect on the researcher's insider/outsider positioning, and triangulate the research findings.

### Data analysis

IPA aims to explore and understand how people make sense of their internal world and lived experiences, by embracing a flexible set of guidelines for data analysis which aims to be “fluid, iterative, and multi-directional” (Noon, 2018, p. 78). Multiple readings of transcripts alongside the audio recordings—to enable as close immersion to the data as possible and generating exploratory notes and experiential statements (Smith et al., 2022)—consisted of two stages. First, a margin was created on the right side of the transcript for exploratory notes which were categorized using different colored highlighters to distinguish between descriptive notes (the content of what the participant was sharing, including specific words and explanations they used that capture the “face value” of their experiences), linguistic notes (capturing the language, tone, pronouns, and degree of fluency), and conceptual notes (the more interpretative and interrogative comments that give insight into the overarching nuances of the experience participants are sharing, which can include questions to help re-analyze other comments and notes to help understand abstract concepts that transcend surface-level descriptions) (Smith et al., 2022, p. 83). Second, a left-hand margin was created for experiential statements which aimed to reduce the volume of notes and to summarize the salient features of each comment and note created. From here, connections were searched for across the experiential statements by creating a separate document with the personal experiential themes and organizing them to recognize patterns in both convergence and divergence across participants’ experiential statements, resulting in a visual representation of group experiential themes linked to subthemes and experiential statements (Table 2).

**Table 2:** Process of creating group experiential themes.

Group experiential themes	Subtheme	Experiential statements
Inescapability and relentlessness of COVID-19	A sitting target: Going to war without weapons.	Physically unprotected by the system – lack of PPE. Emotionally unprotected by the system.
	The intruder invading all aspects of life.	COVID-19 takes priority over personal life. Nursing identity does not stop at home. Concerned about family in Philippines.
	Racism and xenophobia.	Targeted racial assault. Filipino lives are dispensable. Stigmatized for wearing a mask.
It’s sink or swim: Psychological welfare	Overwhelming anxiety, worrying and exhaustion. Facing death is isolating and agonizing. Getting on with it – ways of coping.	The invisible barrier – unable to go home. Overstretched and burnout at work. Mourning without a community Compartmentalizing & minimalizing Spread advocacy and keeping faith Engaging in humor/making light of situations

## Findings

The group experiential themes explored in this paper are *inescapability and relentlessness of COVID* and *“It’s sink or swim”: psychological welfare*, which illuminate the Filipino nurses’ complex and nuanced experiences of being on the front-line of the COVID-19 pandemic, underpinned by cultural, racial, and social factors. The first theme focused on giving insight into participants’ day-to-day experiences of the COVID-19 pandemic, highlighting features that impacted their sense of safety and perpetuated their experience of being the embodiment of threat. The second theme further extrapolates this by demonstrating impacts on participants’ wellbeing and their ways of coping during this distressing and isolating time.

### Theme 1: Inescapability and relentlessness of COVID-19

This theme captured the endless battle on the front line that the nurses had to undertake which permeated from their professional lives into their personal lives. This experience was reinforced by the lack of PPE available to them and being dismissed by superiors within the system, which ultimately created a sense of dispensability and unworthiness of protection.

#### A sitting target: Going to war without weapons

Filipino nurses felt defenseless and exposed, alluding to being on the front line without appropriate PPE was akin to being at war without weapons. This was a particularly novel and jarring experience for them, as they were accustomed to wearing face masks in hospitals in the Philippines. Knowing that other countries had already escalated to mandating higher grade PPE for their HCW led to a feeling of frustration and hopelessness as it meant that the UK was not taking this COVID-19 pandemic as seriously as other countries:

If you’re in the front line, it’s not something you’d be happy to go into because it’s like putting yourself in the war, without the proper weapons, you know, without PPE[…] even though we’ve been escalating our concerns that other countries are also already doing this [higher grade PPE]. Why can't we implement that same thing in the UK? (Megan)

To further compound their feeling of defenselessness, they also expressed that the onus was solely on them to look for PPE in the hopes of protecting themselves:

There wasn’t anything put into place by the infection ward. If there is something that looks suspicious, then they will have to run and look for PPE, like going to ITU [intensive therapy unit] to get PPE. (Karla)

The disparity between how the UK system was protecting their HCW on the front-line, compared to the Philippines’ more proactive approach, left participants feeling confused and unsure of what to believe:

The lack of PPE was a thing. We were just using the plastic apron thingy and masks. It was a bit later they introduced the face shields. I mean it’s weird because, my friends, my counterparts in the Philippines, were wearing these hazmat suits which were covering their entire bodies, and here, we were wearing that plastic apron, gloves, and I thought are the other places overreacting, or is the UK not doing enough? (Carlos)

The use of “it’s weird” to describe how the Philippines had access to hazmat suits and enhanced PPE further emphasized Carlos’ perplexity, as the UK’s medical system is viewed as world-class and advanced, especially by those coming from developing countries. Therefore, this felt like a huge blow to their morale, as their well-being and safety were reduced to the bare minimum, reinforcing the idea that they were dispensable. Further, Anna said,

They were giving us plastic aprons, and it's like, oh my God, this will not protect me at all[…]. You’re still exposed. So, I was thinking, oh my God, we are not prepared for this, and I was just counting down the days until I probably get it.

Participants felt that they had no choice but to accept their fate, as they were made to work on COVID-19-facing wards, despite recently shielding and following multiple attempts to escalate their concerns to their managers around their lack of physical protection; for these participants, reaching out for help was a challenge in itself, likely underpinned by Filipino cultural traits of being subservient to those in higher positions. Karla stressed

Me? Really, me? I have to do that? Can someone else not go there? I really asked if someone else could do it, and they said no one else can go there. It’s only you who can go there. (Karla)

Participants reported that they worked in the COVID-19-facing wards more than their White counterparts, and when advocating for themselves, their nursing professionalism and identity were weaponized against them, demonstrating that these nurses had no choice but to continuously place their lives in danger for the betterment of others.

This was shared by Megan, who advocated for her wellbeing by requesting advanced PPE and when that was not met, to move to non-COVID-19-facing wards:

They said that it's part of your contract that you will comply to whatever sort of job you’re given […] and it's also part of your professionalism, and it’s part of your NMC [Nursing and Midwifery Council] code. You wouldn’t be choosey of the sort of patients you’re going to look after.

Given that they are recruited from the Philippines, many of the participants are reliant on their work visa, which is sponsored by the NHS. As such, the experience of being dismissed for their basic need for protection perpetuated immense power differences between the participants and the system. Affirming this power differential was their nursing counterparts’ ability to refuse work situations where Filipino nurses could not:

The locals would refuse. Like, you could say they’d be given a disciplinary action, but that local won’t have as much to lose, as they can still stay and live in the UK. But for us, if we stop working, we have to go back to our home country because our visa will be terminated, and we won’t get any support from the government if we are made redundant. (Megan)

Megan showcased the collective experience of Filipinos being outsiders and that their value within the UK is tied solely to the work they can provide for the public. Similarly, Carlos felt exploited as he experienced being placed on the COVID-19 facing ward more frequently than his White colleagues:

I just, I just realized that I was put in the Covid base more than my White counterpart. Yeah, I just realized. Gosh, I didn’t care back then, I couldn't say, “I was there yesterday with the Covid people. Can no one else go there?” No, I didn't do that. (Carlos)

By denoting that he “didn’t care back then,” Carlos suggests that he allowed it to happen. And Dominic shared, “When we were asked to stay for three hours or six hours, or whether we want to do another shift after a long day, we just don’t know how to say no. We are perceived as submissive or subservient.” These accounts suggest that participants take responsibility for emulating the characteristics used for exploitation, alluding to a sense of internalized racial and cultural oppression.

Participants also expressed that once higher grade PPE was provided, their fit testing failed multiple times: “it failed on me, and I could still smell and taste the substance they sprayed.” Additionally, Karla was allergic to the masks provided and was told by Occupational Health that they were unable to provide a different brand, as masks were centralized based on the trust:

They said if you know which brand works for you, then get a box each time so you have your own supply. The clinical specialists asked me why I was getting those masks, and I told them it’s the only one I’m not allergic to.

Participants therefore felt that their lives were not worthy of protection. They demonstrated multiple attempts at reaching out to managers to provide adequate PPE that fit them and to request work in different areas of the hospitals, given their vulnerability. Yet, despite their needs not being met, participants continued to work on the front line without adequate protection, for fear of losing their jobs and visas.

#### The intruder invading all aspects of life

The COVID-19 pandemic was the main priority in participants’ lives. As soon as they left the hospital and returned home, participants felt that they were still responsible for other people’s lives; they were expected to support their families physically and emotionally in the Philippines, and, to minimize their own experiences of distress and adversities.

This was exemplified by many of the participants. Anna was diagnosed with cancer at the start of the first wave of the COVID-19 pandemic, but was unable to process the news or share this with her extended family. Carlos worked through physical sickness and cultural and social isolation. And Karla was marginalized for being able to shield as a result of her chronic illness. These participants were unable to place their own physical and mental health needs before their patients and before their family members. Anna shared, “I couldn't react to that because it was in the middle of pandemic and people were actually dying left and right.” Carlos concurred, stating, “I had vertigo. I still went to work because I thought maybe even if my vision is like spinning or I’m dizzy, I can still do the job because I don't want to keep the ward, like, short-staffed.” Conversely, Karla was asked to shield due to her chronic illness, yet felt she had let her colleagues down, attributing this to Filipino cultural traits:

You cannot just sit there thinking that everyone is running around and stressed. When you’re at home [shielding], sitting, thinking about others doing their jobs, that was so difficult for me. Really difficult. It’s a Filipino characteristic that we’re very hardworking and we have that caring mentality.

Participants experienced the COVID-19 pandemic to be ever-present, which was underpinned by a heightened sense of desperation to go back to the Philippines to be with their families during this time of immense uncertainty and distress. Participants simultaneously felt helpless in their ability to provide support to their families, which impacted on their day-to-day functioning, as described by Anna and Carlos:

You worry about it every day. You can’t concentrate on your work because if you're worrying about something else, you try to block it out, but then it's in the news. Everybody's talking about it at work, and it's like everybody knows somebody with it, and everybody knows somebody who died of it or is suffering from it. (Anna)

You're just always preoccupied every second that you do your job. Or on your day off, because you're here, and you know you can't really fly in the Philippines. Because the Philippines, they stopped flights from everywhere. (Carlos)

Further increasing their desperation to be with their families in the Philippines was the powerlessness they experienced as a result of the physical barriers in place that separated them from their loved ones: lockdown measures and strict travel restrictions.

Participants’ biggest fear was their family getting sick with COVID-19, as this meant being unable to be there in person to care for them or being unable to attend their funeral if it came to it. Carlos described an agonizing reflection that he was caring for people in the UK who were not his own, denoting the exploitative system that he was a part of:

I was looking after people who were sick and all that, and thinking what if my family can sick and I’m far, I can't be there. And I’m looking at these people, and I can’t, I won’t be able to look after my family if ever something happens.

Christine concurred, saying that “My greatest worry is if they become really, really sick and you know, you couldn't say goodbye in case they will die.”

Participants’ unfolding concerns about their family members’ health was also underpinned by financial responsibilities, which is a common practice within Filipino culture, particularly held among overseas workers. This ultimately accentuated the pressure participants felt and motivated them to continue working on the front line despite the detrimental impacts they were experiencing:

It will be us who's going to shoulder the money, so we have to support them, and the sad part is there are no more hospitals [in the Philippines]. We were trying to just give them some advice, which is just be proactive, try not to get the COVID because if you catch the virus, it'll be the end for you. (Anna)

#### Racism and xenophobia

Participants experienced targeted abuse because of their race and ethnicity. For many, it was their first time experiencing explicit racism and verbal abuse in the UK, which perpetuated the impact that COVID-19 had on their wellbeing. In particular, participants were targeted by the public and colleagues for their desire to wear face masks and were subject to xenophobic abuse from the general public.

The homogenization of Asians was significantly prevalent; participants felt that they were all generalized to being seen as “Chinese” based on their appearance:

People would think that just because you’re Asian, you’ve got the virus. I don’t know, maybe it’s just their perception of how Asians look like, but they associated me to Chinese. They would say, “You look like you’re Chinese,” but I’m actually not. I look like Chinese, and I have the virus. (Christine)

For some participants, targeted racial abuse occurred within a hospital environment. Carlos, for example, felt particularly disrespected by this occurrence as it had taken place in an environment where he was placing his life at risk for the benefit of others: “We were going back to the flat near the hospital when these teenagers like screamed “Covid!” And it was my first time experiencing actual like being harassed for being Asian, and also in hospital parking lot.” Similarly, Megan feared for her safety, as she was verbally abused in public transport:

When I was on the train, there’s this guy who walked past me and said, “This isn’t the way to China. Go back to your own country”. I couldn’t answer back to him because I was alone. I got really offended by that, but I just didn’t feel like I could do anything in that moment.

While this pattern demonstrated the increase in anti-Asian hate that was occurring, it also provided insight into the invisibility of the Filipino cultural identity, as participants were not being seen as distinctly Filipino. Moreover, participants had to accept the racist comments and move on, as they feared that the consequences of fighting back and retaliating would impede their ability to continue working and staying in the UK.

Differences in mask-wearing etiquette reinforced the embodiment of threat and disease, as participants felt that their desire to wear a mask early on was not welcomed by colleagues and patients. Despite wanting to wear face masks in public spaces such as during their commutes, participants experienced an added layer of fear as they were aware of being seen as virus carriers. Megan described the disparities in mask-wearing etiquette:

In the Philippines, you could take your own initiative to use a mask if you wanted to, just for whatever reason. It doesn't need to be that you aren't feeling well. But here, even if the patient is coughing up, even during the time of COVID, it’s still shameful to be wearing a mask because it's, they're saying that it looks like you are, like, distancing yourself from the patient.

Participants felt unprotected by the system as they were disproportionately at risk on the front line, making them feel more desperate to protect themselves. Yet, when taking the initiative to wear PPE, they had to consider their sense of safety, as they were seen as the physical embodiment of illness and disease.

### Theme 2: “It’s sink or swim”—psychological welfare

Theme 2 gave insight into how participants’ experiences on the front line impacted their psychological wellbeing and their need to survive through the COVID-19 pandemic in the best way they knew.

#### Overwhelming anxiety, worrying and exhaustion

Participants experienced an immense fear of the unknown which was compounded by their lack of appropriate PPE and ever-changing national rules and guidelines. Carlos shared his insight into the chaos and uncertainty that was unfolding on the front line:

It was very unprecedented. It was so unexpected. It was like living in an actual, apocalypse movie, because, and nobody knew what was happening, the treatments were trial and error until they found what works and what doesn’t, uh, a mode of transmission of the thing, how, how quick really was it to get it to get it[…] How difficult, I mean, how easily can you get it. Is this PPE enough?

Carlos depicted the first wave of the COVID-19 pandemic as “unprecedented” and “unexpected.” Describing his experience of being in an “apocalypse movie” which emphasized his intense anticipation of destruction and desolation; while the word “apocalypse” is commonly used to describe catastrophe, it is also used within religious terminology to describe eschatological salvation, envisioning the end of time as we know it. Carlos’ description of “trial and error” processes was spoken at a frantic pace, likely mirroring how he felt being thrust into a mysterious entity that he had no control over, thereby creating panic within himself which was reflected on the front line.

For some participants, relentless anxiety was underpinned by the UK government’s slow administration of protective measures which was lenient compared to many other nations, including the Philippines, where strict lockdowns were being implemented:

Other countries have already started implementing control measures, like, say, for example, closing down your country to minimize movement of people that contributes to the transmission of the virus. And I found that the UK was a bit slow in doing that, and also at the time they were into testing, um, what you call, was “the herd immunity” wasn’t it? They wanted to pilot that. (Megan)

Conversely, participants expressed immense burnout and described having to stretch themselves to cover absences, which perpetuated their experience of exhaustion and fatigue:

It's like every single day now in my unit, there are at least two absences because of this one, and it's come to a point that, yeah, it's burnout. This is burnout because every single day, you have to stretch yourself, and it's not, like, feasible any more. At one point, I have to play like, you know, a teacher, an NA [nursing assistant], and everything. I’m really so tired I sleep on the bus, and then, sometimes, I just wake up and I’m already here. (Anna).

As Anna demonstrates, participants were compelled to continue working in an unsustainable manner, as this was likely entrenched in the intersection of their cultural and nursing identity.

While participants endured the immense distress and anxiety of being on the front line of the COVID-19 pandemic, it was also the evolving restrictions and lockdown that led to exhaustion, as some participants described a strong sense of frustration and fear when restrictions were eased. They did not trust the system that had been enforcing these changes in strategy:

It's still raging on. We've gone through the hard part, but then again, it's still there, because people haven't learned. Or is there something wrong with the system are becoming too relaxed […] So it’s more frustrating now than probably before, because, you know, now what is happening. You know, for me, I’m getting more frustrated. It's more frustrating now that you know what it is. (Anna)

#### Facing death is isolating and agonizing

Each participant experienced an immense emotional burden of being in close proximity to constant death and dying. They experienced a sense of loss when relating to their families; dealing with these feelings during the pandemic was an isolating and agonizing experience, as many were unable to be among their communities during this mourning period due to the physical constraints of lockdown measures and travel restrictions. Carlos shared what it was like to experience constant deaths around him:

It’s like a blur. Like, sometimes, you forget that ever happened. I'm being honest. Sometimes, you forget that it's ever happened. You just got reminded that there was this time it was like crazy and death here and there. Wrapping bodies, counting body bags. My maximum body scrap would be like four in an hour.

The use of the word “blur” alluded to experiencing glimpses of memories of the past and suggested that Carlos was only starting to make sense of this now, after pushing it away for a while, giving insight into how traumatic the experience must have been for him. The use of “my” when describing his maximum body bag count per hour demonstrated the personal impact it had on him, almost to say that the deaths were *on* him. Additionally, it also emphasized the agony he was in for having to reach that number within the hour.

Participants also expressed immense sadness and grief for the Filipino lives lost during the COVID-19 pandemic:

I remember, there was one auxiliary nurse who was 22 years old who died. He came back from a night shift, but he had to stay an extra three hours because the ward was short-staffed. When he came home, his mum, who’s a mental health nurse, said he collapsed and died at home. He was in his early twenties. He was our youngest Filipino healthcare worker to die of COVID in the UK. (Dominic)

Akin to the well-recognized terminology within the Filipino language *kababayan* meaning “fellow Filipinos,” often used when meeting and relating to other Filipinos outside the Philippines, Dominic emphasizes the enormity of this loss felt on a collective level by describing the death of “our youngest Filipino” which impacted fellow Filipinos—*kababayan* across the healthcare sector and beyond.

Similarly, participants also experienced an ongoing mourning process for the Filipino HCW they knew personally. Karla, for example, lost her close friend David, and felt particularly isolated as she was unable to engage in the common Filipino grieving practices, many of which include holding a week-long wake, observing a nine-day period of prayer known as “novena”, and a 40-day mourning process, all of which brings in extended families, friends and neighbours together (face-to-face) to form social congregations and strengthen connections within the community in support of the bereaved (Gamad et al., 2022). This meant that for Karla, it is likely the grieving process felt incomplete and unfinished, due to the changes in funeral practices caused by the pandemic:

I know the ashes of David was taken to the Philippines after two years because of the travel restrictions[…] because no one was allowed to travel, so he had his death anniversary here. His ashes were still here. It’s that kind of feeling.

Conversely, participants questioned their lives and mortality, particularly in relation to how the UK treats Filipinos, and how they would support them after death: “Are they going to cover sending my body back to the Philippines? [laughs] It’s thoughts like that I had.” Carlos’ concern symbolizes the importance of being buried on home soil which perhaps felt especially distant at a time when travel bans were in place. The way in which Carlos conveyed his honest and formidable reflections about his death by making light of the experience alludes to these emotions being overwhelming and difficult to sit with.

#### Getting on with it—ways of coping

Every participant shared their perseverance in overcoming psychological detriments through different means. A common way of coping with emotional distress was to compartmentalize and minimize, make light of the experience, and have faith, all of which are embedded within Filipino cultural norms. Additionally, for some of the participants, supporting others through activism helped alleviate the emotional impact during this time:

I'm usually a resilient person. You know, if I encounter these kinds of difficulties, like, yeah, I’d think to myself this is difficult, but what are you going to do about it? So, I try to condition myself or to face the, the adversity and to survive at that point in time. (Christine)

Christine, like other participants, focused on cultivating resilience and strengths to face adversities by conditioning herself to survive, which gave insight into how ingrained this way of being is within both Filipino and nursing culture. Within Filipino culture, resilience is worn with pride, which, compounded by nursing values, likely increased Christine’s determination to continue prioritizing care for others over herself. Similarly, Anna engaged in compartmentalization to remain calm and avoid spreading panic to loved ones:

You have the responsibility to keep calm and keep going. But deep inside, I was, like, getting really worried because, you know, all the deaths was getting nearer and nearer my work site. So, yeah, I was kind of torn, keeping up this face. [smiles] (Anna)

For other participants, compartmentalizing helped them to accept the reality they were in to move on and deal with the present moment. Christine’s justification of “you win, you lose sometimes” helped her accept the experience of having to constantly face death, expressing that: “I don't dwell on the sad bit. Yeah, I kind of think about it like you win, you lose sometimes.”

It appeared that making light of the situation by laughing, using humor, and changing topics/avoidance helped to mitigate overwhelming emotions and was an important coping mechanism for participants. Anna and Carlos particularly engaged in these strategies:

Yeah, funny enough, I had the same experience before, when I was working in Saudi with the MERS [Middle East Respiratory Syndrome], so I was thinking, yeah, I escaped that one because I got sick then. And then, when I went to the UK, I was thinking, oh my God, it's following me. The coronavirus is following me. [laughs] (Anna)

Carlos agreed: “You just get reminded that life is so fragile that you can just go any time so like, live your life. [laughs] How do you pronounce your last name?” Carlos’ quick change of topic by asking how my last name was pronounced further depicted how difficult it was for him to sit with these overwhelming feelings.

Karla and Dominic spoke of how important it was for them to believe in a faith to guide them throughout the COVID-19 pandemic. Karla felt that attending an online church and praying was the only way to help her through her best friend’s death:

Because you couldn’t go into church, there’s an online mass with this priest, Father John. Almost every day, I was doing that online mass through streaming. I just sat there and listened on my own. When Joe [Karla’s son] did online schooling, I would go upstairs into the room. I couldn’t really do anything else. (Karla)

Karla described the intimate and healing experience of how praying and attending church mass had helped to make sense of the grief and isolation. It felt as though it was the only containing space she could access at the time. Moreover, Dominic shared an insight into the importance of Christianity within Filipino culture, highlighted by his use of “as you know,” emphasizing that this is common knowledge amongst Filipinos:

I think, part of the Filipino culture is being resilient, and, as you know, we have faith in our God. I’m Christian, and I was brought up as a Catholic. But I've got faith in God, and also faith in my colleagues. We trust each other. (Dominic)

Accounts illuminated the detrimental impacts that the COVID-19 pandemic had on the participants’ psychological wellbeing and how participants fought their way through these intense feelings of isolation and fear by compartmentalizing and minimizing their experiences, distracting themselves with day- to-day activities, and, for some, keeping their faith strong and focusing on their advocacy to help guide them through this period.

## Discussion

The themes: *inescapability and relentlessness of COVID* and *“It’s sink or swim”: psychological welfare* captured the sheer overwhelmingness on the front line of the COVID-19 pandemic, demonstrating the severity of the all-consuming and unprecedented entity that was unfolding, impacting all aspects of participants’ lives. Particularly prevalent throughout participants’ narratives was the way they made sense of the intersection of fear and professionalism; the nurses feared for their safety and livelihood which was heightened by a lack of appropriate PPE, yet, ultimately had no choice but to continue working without the protection, placing themselves at continual risk. This experience of threat was felt on multiple levels as the nurses showcased their embodiment of risk from a racial perspective, a public health and political perspective, and on an individual level, capturing the inescapability they were in.

### Mental health impact

Participants alluded to their mental health being severely impacted by the lack of physical protection on the front line, feeling defenseless as they feared not only for their own health and safety, but the wellbeing of their loved ones back in the Philippines. This notion has been explored in previous research, as many studies have demonstrated the mediating factor of PPE availability on the wellbeing of HCW during the COVID-19 pandemic (Sampaio et al., 2020, Wanigasooriya et al., 2020). Additionally, research on this topic highlights the traumatic impacts of working throughout the COVID-19 pandemic (Gilleen et al., 2021, Sampaio et al., 2020, Wanigasooriya et al., 2020), with the focus of using diagnostic labels such as “PTSD” and “Secondary Traumatic Stress” (STS), which do not necessarily capture the universal phenomenon of distress exposure (Hinton and Lewis-Fernández, 2011, Patel et al., 2022). Unlike the aforementioned studies, participants in this study did not use diagnostic terminology to describe their traumatic experiences, which is significant as it illuminates the importance of considering cultural factors and language when conceptualizing mental health. The lack of cultural nuance runs the risk of perpetuating the absence of Filipino nurses’ experiences in extant research on mental health impacts and the development of culturally sensitive mental health interventions. As such, this finding extends previous research into ethnic minority HCWs (Abbas et al., 2020, Hoernke et al., 2021, Moorthy and Sankar, 2020), as it provides rich and nuanced insight into the way Filipino HCWs may mask or normalize their distress and wellbeing, reinforced by years of internalized oppression (Cordova, 1973, David and Okazaki, 2006) and perpetuated by ongoing health and racial inequalities unveiled by the COVID-19 pandemic.

As we have seen, Carlos described counting body bags day after day as a “blur” and alluded to being able to capture only glimpses of his experience working during the first wave of the COVID-19 pandemic; this could be explained by research on trauma responses in relation to dissociation (van der Kolk et al., 1996, Samson and Shvartzman, 2018). “Dissociation” is described as the complex process of detachment and disconnection from oneself and one’s surroundings, which is prevalent within a clinical level among HCW (Samson & Shvartzman, 2018). It appears that participants in this study experienced trauma and dissociation similar to what has been seen in other research (Tsouvelas et al., 2022).

Given that research suggests an already elevated level among HCW prior to the COVID-19 pandemic, distressing and overwhelming firsthand experience on the front line of a global pandemic that was novel and unprecedented would have undoubtedly intensified these trauma responses. As such, these findings generate in-depth insight into the impacts of being seen as both subservient *and* resilient (Guevarra, 2009), while being overrepresented on the frontline of healthcare settings (Nazareno et al., 2021). Participants in the current study illuminated their difficulty of seeking support, as they felt compelled to continue fighting head-on.

### Conceptualizing Filipino cultural traits

Viewed through a western psychology lens, it could be suggested that participants were engaging in adaptive protective factors, as they were resourceful, hopeful and determined to succeed. Moreover, participants engaged in coping strategies which included minimizing, distracting, and making light of situations to help them get through the COVID-19 pandemic. These patterns echo current literature suggesting that nurses experiencing STS, such as work-related distress exposure, also engaged in significant denial, dissociative, and self- distracting coping mechanisms, such as humor, venting, and behavior disengagement (Tsouvelas et al., 2022). However, the current findings reveal a more nuanced picture, by illuminating the cultural underpinnings of these ways of coping, as participants also expressed ingrained perseverance, resilience and “hardiness” that have been attributed to Filipino cultural characteristics (Cleofas et al., 2021, Maiorano et al., 2020). This showcases the importance of embedding the Indigenous approach known as *sikolohiyang Pilipino* (Filipino psychology) (Enriquez, 1975) when exploring the experiences of Filipinos, as cultural protective factors such as collectivistic and family orientation (Ladrido-Ignacio & Versoza, 2011), resourcefulness (Adviento & de Guzman, 2010), engaging in humor and cultivating spiritual empowerment (Alcantara, 2020), help to mitigate perceived vulnerability and impact when facing adversities. Furthermore, it reflects Rilveria’s (2018) Filipino coping scale, as all participants engaged in “problem-solving (*pagtugon*)” particularly around their own health and safety risks which they felt pressured to manage themselves (i.e., social isolation, healthy eating, and mask-wearing in public) as well as predominantly engaging in “tolerance (*pagtitiis*)” by enduring the distress and adversity without having to confront it. Thus, the findings of this study illuminate Indigenous protective factors that helped to mitigate vulnerability within this cohort and emphasizes the importance of integrating intersectionality into the healthcare sector in the UK (Mothupi et al., 2023). This would allow for the development of more inclusive and culturally attuned support, which seemed to be inaccessible for the participants in this research.

### Embodiment of threat and disease

As we have seen, participants experienced overt racism in the UK for the first time, as many of them were targeted in public space and within the workplace. This finding adds to the current literature on anti-Asian hate crimes during the COVID-19 pandemic, as this study captured the intensity of the homogenization of Asians during this period (Wang et al., 2021) and showcased the lived experiences of being a victim of anti-Asian hate crimes in the Western world (Matsuda, 2021, Lim et al., 2022). Moreover, this experience of embodied racial threat was compounded by cultural differences in mask-wearing etiquette, as face masks are viewed as highly stigmatizing in Western cultures (Ma & Zhan, 2022); therefore, participants felt that their safety was out of their control, as they were perceived as a threat to the UK public.

Current research also adds to the body of literature by demonstrating important factors to consider in the hope of making Filipino nurses feel safe and accepted within their workplace and able to have their needs met by colleagues and managers. Watson et al. (2020) and Maben and Bridges (2020) provided recommendations for the wellbeing of HWCs during the start of the COVID-19 pandemic which included addressing physical needs first and subsequently meeting safety needs on an individual, peer-to-peer, and team-level; however, their suggestions do not take into account cultural factors that inform the way different needs are expressed or the various aspects of safety that ethnic minority HCWs need.

These findings illuminated the need to make space for and to include non-dominant cultural discourses within the workplace, particularly when making decisions that are meant to mitigate risk. Participants highlighted their need to feel accepted, for the personal use of face masks as a preventive measure, and to be heard when concerns were raised around the incorrect and ill-fitting PPE.

#### Limitations

While this research study has many notable strengths, there are some limitations including sample demographics and method of recruitment. The scope of recruitment focused predominantly on social media platforms such as Facebook, Instagram, and LinkedIn. Also, participants’ pay bands^2^ were not considered. Therefore, we may have missed out on a cohort of potential nurses who were not on social media and those who held jobs across different pay bands who may have experienced COVID-19 differently from these six participants.

#### Implications and future directions

Participants' narratives shared in this paper demonstrate the determination to persevere and push through moments of prolonged distress with minimal emotional and physical support from their workplaces, which was detrimental to their wellbeing. These findings suggest that to safeguard Filipino HCWs—which is ultimately important for retention rates—providing emotional support and containment within colleague-to-colleague and colleague-to-manager levels where one can share and reflect on thoughts, feelings, and concerns. Moreover, while this is consistent with current recommendations made by Maben and Bridges (2020), this study further emphasizes the importance of management to act on concerns for meeting physical needs, such as providing PPE that fits smaller and shorter features,^3^ and comprehensively engaging in ongoing risk-assessments to mitigate risk.

This research demonstrates the masking and normalizing of distress amongst Filipino HCWs, which the UK health system has overlooked. With these settings, management must be aware that Filipinos are more likely to be taken advantage of than their White colleagues as a result of their cultural traits such as resilience, agreeableness, and compliance. Therefore, supervisors and managers should be aware when Filipinos are taking on extra shifts with minimal rest in between and should reflect if they are asking Filipinos to place themselves at disproportionate levels of risk. Moreover, when working with Filipino HCWs in a therapeutic capacity, the findings suggest the importance of avoiding psycho-pathologizing or medicalized terminology concerning their mental health, for example using terms as “depression”, “trauma” or “anxiety”, and instead focusing on phenomenological and embodied aspects of experiencing, as it allows the Filipinos to make sense of their well-being and within their own terms, which, ultimately provides a more culturally inclusive safe space. Further, we acknowledge that this may be applicable to other minoritized staff who may also have been impacted by the lack of parity in the workplace, as evidenced in previous research (Abbas et al., 2020, Hoernke et al., 2021, Moorthy and Sankar, 2020).

Lastly, this study highlights the importance of working towards more culturally sensitive formulation models through evoking personal and professional reflection and encouraging more discussions around inclusivity in the workplace, in social contexts, and clinical settings. This could include training for staff to understand cultural differences in models of care and treatment, such as incorporating Considerations of Cultural Sensitivity for Healthcare Providers (Ayonrinde, 2003), the Cultural Competency Model (Papadopoulus et al., 2016), and the Power Threat Meaning Framework (Johnstone et al., 2018) as ways to enhance knowledge and sensitivity of different cultures and to learn about the importance of power structures and varying levels of oppression that may unfold in the workplace.

Future research could specifically explore this phenomenon through the lens of intersectionality, as this would provide more space for the marginalized and racialized voices within the Filipino HCW community and generate further insight into levels of oppression which would be important to establish for a policy standpoint. Additionally, there was a striking difference in how participants in higher pay bands related to themselves, others, and their work, during the COVID-19 pandemic. Given that, just over one percent of Filipino HCWs in the NHS are at a Band 8 or above and that London has been identified as having a higher disparity ratio for staff progression into higher bands (8A above) for ethnically marginalized staff (NHS England, 2022), it would be significant to account for these pay bands in future studies, exploring the experiences of senior managerial Filipino HCWs and their career journey. This may provide further insight into fellow Filipino HCWs, and task services to ensure better inclusion of ethnic minority backgrounds in regard to their career opportunities and progression.

## CRediT authorship contribution statement

**Borbolla Denise:** Conceptualization, Data curation, Formal analysis, Methodology, Visualization, Writing – original draft, Writing – review & editing. **Nkansa-Dwamena Ohemaa:** Supervision, Writing – review & editing.

## Author statement

The authors confirm sole responsibility for the following: study conception and design, data collection, analysis and interpretation of results, and manuscript preparation.

## Declaration of interests

The authors declare the following financial interests/personal relationships which may be considered as potential competing interests: Denise Borbolla - I was a trainee Counseling Psychologist during this time and worked in the NHS for clinical placement, although I did not recruit directly in the NHS nor did I receive any funding for this research. Ohemaa Nkansa-Dwamena - I am a counselling psychologist and associate professor who supports trainees working within the NHS. I did not receive any funding for this project.

## Footnotes

1 The UK's National Health Service (NHS) has several organizational units called “trusts” that provide healthcare to the local population. These trusts can serve a specific function such as an ambulance service and mental health services or serve a geographical area.

2 The NHS pay system covers all staff except doctors, dentists, and very senior managers. There are nine pay bands which have several pay points. Staff will normally progress to the next pay band after the required number of years until they reach the top of the pay band. Pay bands start at 1: £23,615 and end at 9: £121,271 (NHS Agenda for Change).

3 Facial dimensions for the fit-test of medical masks are often tested from a cohort in which female and Asian demographics are underrepresented (Chopra et al., 2021).
